# Metabolic remodeling during somatic cell reprogramming to induced pluripotent stem cells: involvement of hypoxia-inducible factor 1

**DOI:** 10.1186/s41232-020-00117-8

**Published:** 2020-05-12

**Authors:** Tomoaki Ishida, Shu Nakao, Tomoe Ueyama, Yukihiro Harada, Teruhisa Kawamura

**Affiliations:** 1 Department of Biomedical Sciences, College of Life Sciences, Ritsumeikan University, Kusatsu, Japan; 2 Ritsumeikan Global Innovation Research Organization, Ritsumeikan University, Kusatsu, Japan

**Keywords:** Induced pluripotent stem cells, Reprogramming, Metabolic shift, Hypoxia-inducible factor, Regenerative medicine, Glycolysis, Oxidative phosphorylation

## Abstract

Induced pluripotent stem cells (iPSCs) were first established from differentiated somatic cells by gene introduction of key transcription factors, OCT4, SOX2, KLF4, and c-MYC, over a decade ago. Although iPSCs can be applicable for regenerative medicine, disease modeling and drug screening, several issues associated with the utilization of iPSCs such as low reprogramming efficiency and the risk of tumorigenesis, still need to be resolved. In addition, the molecular mechanisms involved in the somatic cell reprogramming to pluripotency are yet to be elucidated. Compared with their somatic counterparts, pluripotent stem cells, including embryonic stem cells and iPSCs, exhibit a high rate of glycolysis akin to aerobic glycolysis in cancer cells. This is known as the Warburg effect and is essential for maintaining stem cell properties. This unique glycolytic metabolism in iPSCs can provide energy and drive the pentose phosphate pathway, which is the preferred pathway for rapid cell proliferation. During reprogramming, somatic cells undergo a metabolic shift from oxidative phosphorylation (OXPHOS) to glycolysis trigged by a transient OXPHOS burst, resulting in the initiation and progression of reprogramming to iPSCs. Metabolic intermediates and mitochondrial functions are also involved in the epigenetic modification necessary for the process of iPSC reprogramming. Among the key regulatory molecules that have been reported to be involved in metabolic shift so far, hypoxia-inducible factor 1 (HIF1) controls the transcription of many target genes to initiate metabolic changes in the early stage and maintains glycolytic metabolism in the later phase of reprogramming. This review summarizes the current understanding of the unique metabolism of pluripotent stem cells and the metabolic shift during reprogramming, and details the relevance of HIF1 in the metabolic shift.

## Background

Induced pluripotent stem cells (iPSCs) are established by the introduction of reprogramming factors (OCT4, SOX2, KLF4, and c-MYC) into somatic cells. Over a decade ago, Takahashi and colleagues reported that iPSCs can be generated from mouse and human somatic cells, and that they exhibited the ability to replicate identically (self-renewal) and differentiate into any cell types except extraembryonic tissues (pluripotency), as observed in embryonic stem cells (ESCs) [1, 2]. iPSCs currently hold promise for application in regenerative medicine, disease modeling, and drug screening [3, 4]. However, the risk of oncogenicity and low reprogramming efficiency interfere with the realization of the practical applications of iPSCs [4–6]. To solve the above mentioned problems, multiple approaches have been tested by modifying the combination of introduced reprogramming genes or using miRNAs and synthetic mRNAs, some of which result in 90–100% the reprogramming efficiency [7, 8]. Nevertheless, it is critical to elucidate the molecular mechanisms underlying the reprogramming of somatic cells to iPSCs. During reprogramming, somatic cells undergo transitions in gene expression profile, epigenetic status, metabolic characteristics, and cellular morphology [9, 10]. The importance of metabolic remodeling during the reprogramming to pluripotency has been proposed in many studies over the last decade [11–15]. The predominance of metabolism transits from oxidative phosphorylation (OXPHOS) to glycolysis has been observed during reprogramming. Glycolysis is a process with relatively low efficiency in terms of energy production; however, if the rate of metabolic flux is high enough, glycolysis could produce sufficient ATP for rapid cell proliferation. It is also important to note that glycolysis is essential for the biosynthesis of nucleic acids, amino acids, and lipids [16, 17]. Interestingly, this aerobic glycolysis is similar to the metabolic property observed in cancer cells and other types of stem cells such as ESCs [11, 17]. This metabolic character, known as the Warburg effect, is promoted by hypoxia-inducible factor 1 (HIF1), a core regulator of aerobic glycolysis [18]. HIF1α, the main subunit of HIF1, is rapidly degraded under normoxic conditions. However, under the hypoxic conditions found within a tumor tissue, HIF1α is stabilized and regulates the transcription of its many target genes that contribute to tumor growth through increased cell proliferation and neovascularization [18]. HIF1 also promotes glycolysis by direct transcriptional regulation of glycolytic genes that switches the metabolic flux from OXPHOS to glycolysis. To date, several studies have investigated how the metabolic shift to glycolysis occurs in the reprogramming process, and they have demonstrated the importance of HIF1 in the metabolic shift. In this review, we summarize the current understanding of the dynamic changes in metabolic properties in the process of somatic cell reprogramming to iPSCs, with a special focus on the role of HIF1 in metabolic remodeling as a reprogramming enhancer.

### Metabolic features of PSCs

The metabolic features of iPSCs are similar to those of ESCs, which are characterized by high glycolysis flux accompanied by low OXPHOS flux for ATP production as mentioned above [12, 19–23]. A similar metabolic phenomenon, called the Warburg effect, is observed in cancer cells [17, 24]. It is known that cancer metabolic processes are heterogeneous and vary depending on intratumoral oxygen concentrations. Particularly in hypoxic conditions, cancer cells generate ATP and metabolites for constructing cellular components through aerobic glycolysis regulated by a HIF1-dependent mechanism [25–27], because intermediate metabolites in glycolysis and pentose phosphate pathways are required for high proliferative growth. Intermediate metabolites in glycolysis are also involved in stem cell properties. Several studies have revealed that glycolytic metabolism in ESCs is regulated by multiple mechanisms. For example, in the first step of glycolysis, hexokinase (HK) catalyzes phosphorylation of glucose to yield glucose-6-phosphate (G6P). Later, pyruvate kinase (PK) converts phosphoenolpyruvate to pyruvate. These glycolytic enzymes are expressed at a higher level in ESCs than in somatic cells. Increased activity of HK2, an isoform of HK, and PKM2, an isoform of PK, maintains a high glycolytic rate in ESCs that contributes to pluripotency even in the absence of leukemia inhibitory factor (LIF) [28]. On the other hand, the inhibition of HK2 by 3-bromopyruvate causes a metabolic change from glycolysis to OXPHOS, leading to loss of pluripotency even in the presence of LIF [15, 28, 29]. Inactivation of pyruvate dehydrogenase (PDH) also maintains high glycolytic metabolism in ESCs [15]. In addition, core reprogramming factors play a critical role in sustaining high glycolytic flux. OCT4 is known to directly regulate HK2 and PKM2 transcription in ESCs [28]. Conditional double knockout of *c-Myc* and *N-Myc* in ESCs impairs self-renewal and pluripotency associated with downregulation of genes involved in cellular metabolism [30]. Conversely, inhibition of glycolysis using 2-deoxyglucose leads to the loss of pluripotency of PSCs [31]. Moreover, cell proliferation requires ATP and metabolic intermediates from the pentose phosphate pathway. In highly proliferative cancer cells, glycolysis provides G6P to the pentose phosphate pathway to generate ribose 5-phophate for nucleotide biosynthesis [16, 32]. ESCs and iPSCs also utilize these metabolic intermediates for rapid cell proliferation and pluripotency [12, 15, 21, 29]. Several studies have demonstrated that upregulation of genes involved in glycolysis and pentose phosphate pathways results in epigenetic changes in the early stage of iPSC reprogramming [12, 15, 22].

Despite the relatively low contribution of OXPHOS to ATP production in PSCs compared to that in somatic cells, mitochondria still play an important role in biosynthesis of metabolic intermediates [33]. Aerobic glycolysis in cancer cells is similar to that in PSCs, but not exactly same. In glycolysis, cancer cells use pyruvate to generate lactate, whereas in PSCs, glucose is increasingly converted to acetyl-coenzyme A (acetyl-CoA) [33]. Subsequently, acetyl-CoA is converted to citrate by citrate synthase in the mitochondria. Citrate is then exported from the mitochondria to the cytoplasm and is again converted to cytosolic acetyl-CoA by ATP-citrate lyase. Cytosolic acetyl-CoA acetylates histones in ESCs to maintain the open state of the chromatin structure, leading to pluripotency maintenance in PSCs [33, 34]. In contrast, the loss of acetyl-CoA results in histone deacetylation and loss of pluripotency in PSCs [33]. Acetyl-CoA, together with glycine produced in the threonine metabolism pathway, is also essential for S-adenosylmethionine (SAM) synthesis [35]. SAM contributes to histone methylation, for example, H3K4me3, leading to the pluripotency of “naïve” mouse ESCs. In addition, human “primed” PSCs utilize methionine to generate SAM and maintain H3K4me3 levels for the maintenance of the pluripotency [36]. In terms of the amino acid utility, human primed PSCs also rely on glutamine oxidation to synthesize ATP [37]. Even in the absence of the glycolytic flux, primed PSCs can survive through nucleotides and glutathione synthesis and energy production via the glutamine metabolic pathway [37]. In contrast, glutamine depletion results in a decrease in αKG levels leading to histone methylation and subsequent differentiation [38]. However, in naïve PSCs, the absence of glutamine does not affect cell proliferation and histone methylation when glucose is available as an energy source [38]. This might be because the origin of naïve and primed PSCs is different. The former is established from the inner cell mass of the blastocyst, while the latter is derived from the post-implantation epiblast where there is no blood supply in utero [39, 40].

**Fig 1:**
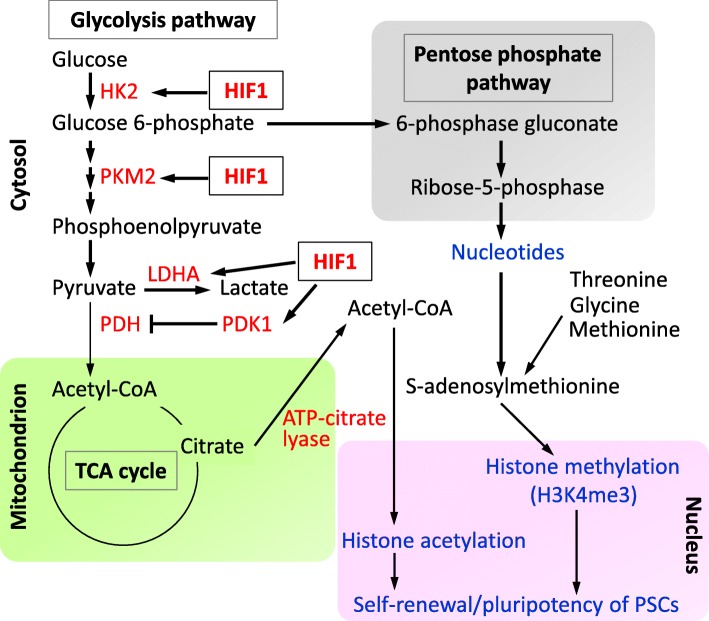
Glycolytic metabolism and its functional relevance in PSCs. Acetyl-CoA, acetyl-coenzyme A; ATP, adenosine triphosphate; HIF1, hypoxia-inducible factor 1; HK2, hexokinase 2; LDHA, lactate dehydrogenase; PDH, pyruvate dehydrogenase; PDK1, pyruvate dehydrogenase kinase 1; PKM2, pyruvate kinase M2; PSC, pluripotent stem cell

The mitochondrial electron transport chain (ETC) is fully functional in ESCs. Uncoupling protein 2 (UCP2) in ESCs shifts from OXPHOS to glycolysis by shunting pyruvate out of mitochondria [41]. UCP2 uncouples glucose oxidation from ATP production in ETC, thereby reducing reactive oxygen species (ROS) generation. As excessive ROS have the potential to damage nucleic acids, lipids, and proteins in cells, ROS production needs to be maintained at a low level possibly by UCP2 for stem cell maintenance [22, 42]. Therefore, mitochondrion is a critical subcellular organelle that maintains PSC properties through UCP2-dependent modulation of OXPHOS flux for ROS reduction as well as increasing the biosynthesis of metabolic intermediates for epigenetic modification. In addition, HIF1 is involved in the transcriptional regulation of several glycolytic genes that enhance the metabolic flux of glycolysis. Figure 1 shows a schematic illustration of glycolytic metabolism and the involvement of HIF1.

### Metabolic shift during reprogramming to iPSCs

As described, iPSCs predominantly utilize glycolysis to generate ATP and intermediates that contribute to pluripotency and rapid cell proliferation. A metabolic shift from OXPHOS to glycolysis occurs in somatic cells undergoing reprogramming into iPSCs [14, 15, 22]. Several recent studies have investigated the mechanism underlying the metabolic shift during the reprogramming. Glycolytic genes are upregulated early before the induction of pluripotency genes, and they remain upregulated during reprogramming [12, 13, 43, 44]. Glucose uptake and lactate production are constantly increased by upregulation of the glucose transporter GLUT1 and lactate dehydrogenase A (LDHA), which is consistent with the gradual metabolic shift from OXPHOS to glycolysis [12]. Moreover, activation of glycolysis results in improved reprogramming efficiency, whereas inhibition of glycolysis reduces reprogramming efficiency [12, 14, 15, 22].

**Fig 2:**
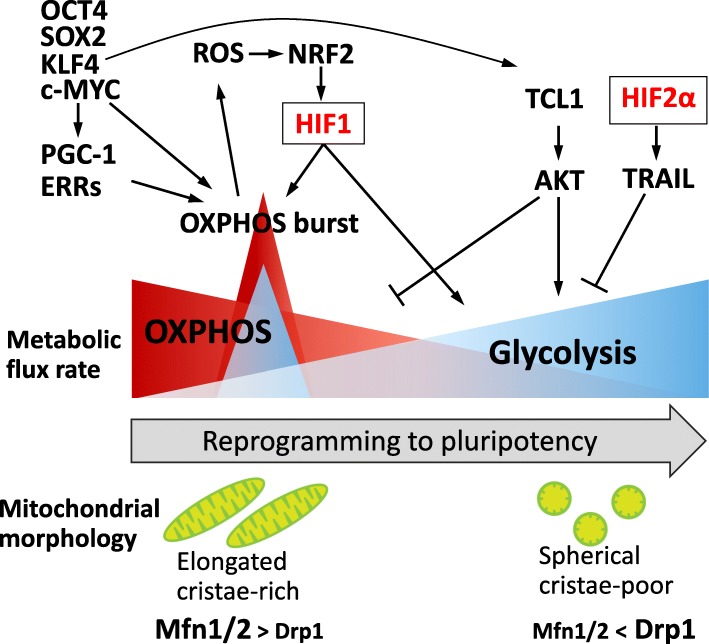
Metabolic shift and its related molecules during reprogramming to pluripotency. DRP1, dynamin-related protein 1; ERRs, estrogen-related nuclear receptors; HIF, hypoxia-inducible factor; Mfn, mitofusin; NRF2, nuclear factor erythroid 2-related factor 2; OXPHOS, oxidative phosphorylation; PGC-1, peroxisome proliferator-activated receptor gamma coactivator-1; ROS, reactive oxygen species; TRAIL, TNF-related apoptosis-inducing ligand

Pyruvate dehydrogenase kinase 1 (PDK1) is one of metabolic enzymes involved in aerobic glycolysis activation. PDK1 phosphorylates and inactivates the PDH complex, which catalyzes pyruvate into acetyl-CoA [45]. As the PDK1 protein is more stabilized in PSCs than in differentiated somatic cells, possibly via a HIF-dependent regulation, reprogramming cells may show enhanced glycolytic activity [15] (Fig. 1). In addition, AKT activity is correlated with the upregulation of glycolytic genes and increases lactate production, resulting in improvement of the reprogramming efficiency [46, 47]. TCL1, a protooncogene that phosphorylates AKT as a co-activator, is upregulated by KLF4 through direct transcriptional regulation. This increased expression of TCL1 was observed in late-stage reprogramming [48, 49]. TCL1 also reduces OXPHOS flux by inhibiting mitochondrial polynucleotide phosphorylase. TCL1 thus promotes metabolic shift in an AKT-dependent manner that increases the reprogramming efficiency [48, 50] (Fig. 2).

Despite the importance of glycolysis in reprogramming to iPSCs, Kida et al. revealed that OXPHOS is transiently increased at the early stage of reprogramming, at which mitochondrial proteins are upregulated [51]. This OXPHOS burst leads to the metabolic shift and is ultimately required for the establishment of induced pluripotency. Mechanistically, estrogen-related nuclear receptors (ERRα and ERRγ) and their co-activators, PGC-1α and PGC-1β, are upregulated transiently during early-stage reprogramming triggered by the introduction of reprogramming factors, particularly c-MYC. PGC-1/ERRs subsequently induce the hyper-energetic state of metabolism necessary for iPSC production. In contrast, the inhibition of ERR expression fails to induce successful reprogramming [51]. Moreover, the fact that c-MYC can significantly upregulate the expression levels of glycolysis- and OXPHOS-related enzymes demonstrates the deep involvement of c-MYC in metabolic shift at the early stage of reprogramming [52]. Considering that c-MYC, together with HIF1, regulates metabolic remodeling in cancer cells under hypoxic conditions [53, 54], c-MYC and HIF1 may cooperate to induce metabolic shift during iPSC reprogramming as well. Hawkins et al. similarly reported that OXPHOS burst during early reprogramming is regulated by nuclear factor erythroid 2-related factor 2 (NRF2) via the induction of HIF1-mediated glycolytic shift and glucose redistribution to the pentose phosphate pathway [55]. In contrast, inhibition of NRF2 by an E3 ubiquitin ligase adaptor, KEAP1, overexpression, or knockdown of NRF2, reduces the reprogramming efficiency [55, 56]. Moreover, prior to NRF2 upregulation, ROS production was increased presumably as a result of elevated mitochondrial activity associated with OXPHOS burst. As NRF2 is known as a master regulator of the antioxidant response, it may protect cells from oxidative stress during reprogramming [55]. Although it remains unclear how the transient hyperenergetic state is decreased to the proper level, OXPHOS burst is essential to initiate and ensures the progress of reprogramming to pluripotency.

Mitochondrial morphology differs significantly between somatic cells and iPSCs. Introduction of the reprogramming factors induces regression from mature tubular and cristae-rich mitochondria to immature spherical and cristae-poor mitochondria. Mitochondrial mass is also decreased by iPSC reprogramming [12]. Forced expression of c-MYC affects mitochondrial morphology, which changes from tubular to fragmented, accompanied by upregulation of the mitochondrial fission regulator, dynamin-related protein 1 (DRP1), and elevation of the mitochondrial membrane potential as a mitochondrial function indicator [52]. As for the effect of modulation of mitochondrial dynamics, DRP1 inhibition reduces reprogramming efficiency [57, 58]. In contrast, deficiency of mitofusin 1 and 2, the mitochondrial fusion regulators, facilitates metabolic conversion to glycolysis and promotes iPSC production [59]. Thus, mitochondria remodeling occurs during reprogramming and influences the efficacy of iPSC production [22, 23, 60]. As discussed so far, these dynamic changes in mitochondrial structure during reprogramming may be highly correlated to mitochondrial function such as OXPHOS and ETC activity, linking to metabolic shift that promotes the acquisition of pluripotency. Figure 2 illustrates the metabolic shift and its associated regulatory molecules during reprogramming to pluripotency.

### HIF1 for metabolic reprogramming

**Fig 3:**
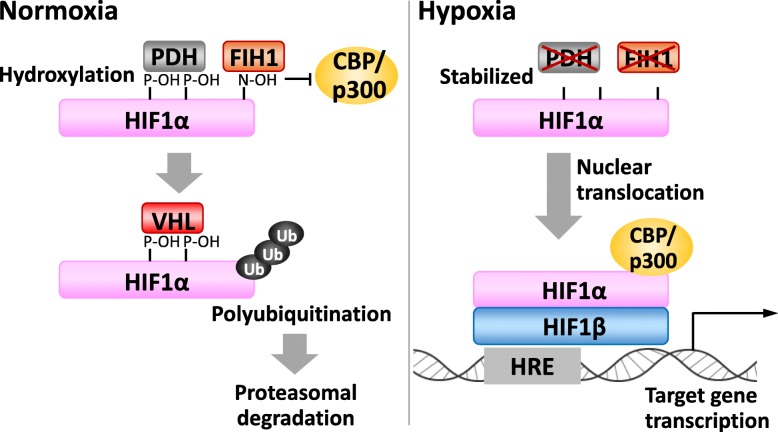
HIF1α turnover and its transcription function. FIH1, factor inhibiting HIF1; HIF, hypoxia-inducible factor; HRE, HIF-responsive element; PHD, prolyl hydroxylase; VHL, von Hippel-Lindau protein

As mentioned above, PSCs predominantly utilize glycolysis for ATP generation, akin to the cancer cell metabolism. Cancer cells utilize glycolysis for ATP production regardless of oxygen conditions; this aerobic glycolysis is known as the Warburg effect [17, 24]. It is known that the primary regulator of the Warburg effect is HIF1 [18, 61]. HIF1 regulates gene transcription of over 100 genes controlling cell metabolism, survival, motility, angiogenesis, hematopoiesis, and other cellular functions in response to hypoxia. HIF1 functions as a heterodimer comprising HIF1α and HIF1β subunits, and the stability of the HIF1α protein depends on oxygen concentration. Under normoxic conditions, proline residues of HIF1α are hydroxylated by prolyl hydroxylase (PHD). Hydroxyprolines allow the binding HIF1α with the von Hippel-Lindau protein, resulting in ubiquitination by an E3 ubiquitin ligase and subsequent degradation by the proteasome [62]. Under hypoxic condition, HIF1α escapes from hydroxylation by PHD because PHD activity is repressed by insufficient oxygen concentrations. HIF1α then accumulates and dimerizes with HIF1β. The dimerized HIF1 then drives the transcription of hypoxia-responsive genes (Fig. 3). Factor inhibiting HIF1 (FIH1), similar to the PHD family members, is a Fe^2+^- and 2-oxoglutrate-dependent dioxygenase. In normoxia, FIH1 hydroxylates an asparagine residue of HIF1α that prevents the transcription co-activators p300 and CBP recruitment. Although FIH1 is less sensitive to hypoxia than PHD, hypoxia inactivates FIH1, allowing further tuning of the transcriptional activity of HIF1 by p300/CBP (Fig. 3).

It was previously reported that hypoxia promotes iPSC generation [63], and an HIF family member, HIF2α, which is also hypoxia-activated and dimerizes with HIF1β, improves the reprogramming efficiency by suppressing the tumor suppressor p53 [64]. More recent reports have demonstrated that HIF family plays a critical role in the metabolic shift during reprogramming. HIF1α increases the expression of glycolytic genes and promotes glycolysis during reprogramming, whereas the knockdown of HIF1α and HIF2α negatively regulates reprogramming [13, 43]. Mathieu et al. showed that HIF1α and HIF2α are stabilized under normoxic conditions during reprogramming, and both HIF1α and HIF2α are required to initiate the metabolic shift at the early stage of reprogramming. However, prolonged HIF2α activation represses iPSC formation, while prolonged HIF1α activation increases the reprogramming efficiency [13]. HIF2α activation at the late stage of reprogramming may repress reprogramming through TNF-related apoptosis-inducing ligand (TRAIL). TRAIL inhibits iPSC formation by repressing apoptotic caspase 3 activity, and inhibition of TRAIL activity enhances iPSC generation [13] (Fig. 2). Therefore, it is suggested that HIF1α and HIF2α have stage-dependent distinctive roles in promoting iPSC reprogramming. Furthermore, Prigione et al. demonstrated that HIF1α promotes the reprogramming efficiency through upregulation of metabolic enzymes PK and PDK, promoting the metabolic shift to glycolysis [43]. They also confirmed that the depletion of HIF1α significantly decreased iPSC formation. Mechanistically, the expression of PKM2, PDK1, and PDK3 is upregulated by HIF1 activation during reprogramming, while the knockdown of HIF1α prevents an increase in the expression levels of these glycolytic enzymes. Moreover, the introduction of reprogramming factors upregulates the expression of PDK1 [43]. These studies thus indicate that HIF1α and HIF2α play a pivotal role in the metabolic shift to glycolysis at the early stage, and that HIF1α maintains a high glycolysis flux in the later phase of reprogramming to pluripotency.

Gene introduction of the reprogramming factors elevates HIF1 activity that promotes iPSC production. Taking the above into consideration, pharmacological stabilization of HIF1α may enable to promote the successful reprogramming even in normoxic conditions. In this context, there are PHD inhibitors known as HIF1α stabilizers that are commercially available for the treatment of renal anemia through promoting the biosynthesis of erythropoietin, one of the common HIF1 target genes, encoding a hormone that stimulates blood production [65]. Thus, although a HIF1α stabilizer may have the potential to trigger tumorigenicity via HIF1-mediated mechanisms [18], it might be of importance to utilize HIF1α stabilizers for efficient production of iPSCs.

## Conclusion

iPSCs are a promising cell source for regenerative medicine, disease modeling, and drug discovery. However, the molecular basis of the iPSC reprogramming process remains not fully uncovered. Recent studies have highlighted the importance of the metabolism of iPSCs, characterized by glycolysis activation, compared to that of its somatic counterparts. Glycolytic metabolism provides ATP and drives the pentose phosphate pathway, contributing to rapid cell proliferation. Despite their lower contribution to OXPHOS in iPSCs than in somatic cells, mitochondria in iPSCs are also highly involved in the acquisition and maintenance of induced pluripotency by providing metabolic intermediates required for epigenetic modifications as well as by reducing ROS production. In the early stage of reprogramming, a transient increase in the OXPHOS rate is an essential event. This OXPHOS burst then declines that reduces the ROS production, followed by sustained glycolysis activation. We also introduced HIF1α, which activates the transcription of many target genes to promote the glycolysis-shifted metabolism necessary for successful reprogramming. In this context, activation of HIF1 by pharmacological stabilizers could be useful for efficient production of iPSCs. Further investigations of the interplaying networks of transcriptional, epigenetic, and metabolic properties in iPSC reprogramming will lead to significant progress in understanding the detailed mechanisms of cell plasticity.

## Data Availability

Not applicable

## Abbreviations

Acetyl-CoA: Acetyl-coenzyme A
ATP: Adenosine triphosphate
DRP1: Dynamin-related protein 1
ERR: Estrogen-related nuclear receptor
ESC: Embryonic stem cell
ETC: Electron transport chain
FIH1: Factor inhibiting HIF-1
G6P: Glucose-6-phosphate
GLUT4: Glucose transporter 4
HIF: Hypoxia-inducible factor
HK: Hexokinase
iPSC: Induced pluripotent stem cell
LDHA: Lactate dehydrogenase A
LIF: Leukemia inhibitory factor
NRF2: Nuclear factor erythroid-related 2 factor 2
OXPHOS: Oxidative phosphorylation
PDH: Pyruvate dehydrogenase
PDK: Pyruvate dehydrogenase kinase
PGC-1: Peroxisome proliferator-activated receptor gamma coactivator-1
PHD: Prolyl hydroxylase
PK: Pyruvate kinase
ROS: Reactive oxygen species
SAM: S-adenosylmethionine
TRAIL: TNF-related apoptosis-inducing ligand
UCP2: Uncoupling protein 2
